# Blending Gelatin and Cellulose Nanofibrils: Biocomposites with Tunable Degradability and Mechanical Behavior

**DOI:** 10.3390/nano10061219

**Published:** 2020-06-22

**Authors:** Elisabetta Campodoni, Margherita Montanari, Samuele M. Dozio, Ellinor B. Heggset, Silvia Panseri, Monica Montesi, Anna Tampieri, Kristin Syverud, Monica Sandri

**Affiliations:** 1 Institute of Science and Technology for Ceramics-National Research Council (CNR), 48018 Faenza, Italy; margherita.montanari@istec.cnr.it (M.M.); samuelem.dozio@gmail.com (S.M.D.); silvia.panseri@istec.cnr.it (S.P.); monica.montesi@istec.cnr.it (M.M.); anna.tampieri@istec.cnr.it (A.T.); 2 RISE PFI, NO-7491 Trondheim, Norway; ellinor.heggset@rise-pfi.no; 3 Department of Chemical Engineering, Norwegian University of Science and Technology (NTNU), NO-7491 Trondheim, Norway

**Keywords:** soft-tissue, polymer blends, nanocellulose, gelatin, cell-tissue interaction

## Abstract

Many studies show how biomaterial properties like stiffness, mechanical stimulation and surface topography can influence cellular functions and direct stem cell differentiation. In this work, two different natural materials, gelatin (Gel) and cellulose nanofibrils (CNFs), were combined to design suitable 3D porous biocomposites for soft-tissue engineering. Gel was selected for its well-assessed high biomimicry that it shares with collagen, from which it derives, while the CNFs were chosen as structural reinforcement because of their exceptional mechanical properties and biocompatibility. Three different compositions of Gel and CNFs, i.e., with weight ratios of 75:25, 50:50 and 25:75, were studied. The biocomposites were morphologically characterized and their total- and macro- porosity assessed, proving their suitability for cell colonization. In general, the pores were larger and more isotropic in the biocomposites compared to the pure materials. The influence of freeze-casting and dehydrothermal treatment (DHT) on mechanical properties, the absorption ability and the shape retention were evaluated. Higher content of CNFs gave higher swelling, and this was attributed to the pore structure. Cross-linking between CNFs and Gel using DHT was confirmed. The Young’s modulus increased significantly by adding the CNFs to Gel with a linear relationship with respect to the CNF amounts. Finally, the biocomposites were characterized in vitro by testing cell colonization and growth through a quantitative cell viability analysis performed by 3-(4,5-dimethylthiazol-2-yl)-2,5-diphenyltetrazolium bromide (MTT) assay. Additionally, the cell viability analysis was performed by the means of a Live/Dead test with Human mesenchymal stem cells (hMSCs). All the biocomposites had higher cytocompatibility compared to the pure materials, Gel and CNFs.

## 1. Introduction

The difficulties and limitations of conventional tissue engineering (TE) have recently given rise to a new concept known as “in situ TE”, where the own regenerative capability of the body is exploited and addressed to enable the regeneration and healing of the target tissue [1,2]. In recent years, the new far reaching goal of the most innovative regenerative approaches for tissue engineering is to restore the original functionality of the damaged or injured tissues and organs, in order to obtain a complete recovery [3]. In this perspective, natural polymers are attracting more and more interest as scaffolds, due to their biocompatibility and when degraded, resorbable nature. The natural polymers can be used either alone or as blends of two or more, i.e., as biocomposites. The scaffolds should be able to support cell adhesion, migration and growth, and to influence cellular functions and direct “in situ” stem cells differentiation. This can be influenced by the scaffold’s chemical composition, stiffness, surface topography, geometry, and permeability. With the advancement of science in regenerative medicine, many efforts are directed to design smart bio-composites able to recruit cells directly on site and instruct them towards the different and complex tasks occurring within the regenerative cascade [4,5,6,7].

In the present work, two different natural polymers, gelatin (Gel) and cellulose nanofibrils (CNFs), were used for the creation of a new family of three-dimensional (3D) porous biocomposites for “in situ” soft-tissue regeneration. The biocomposites were prepared by blending Gel with CNFs in different weight ratios, specifically Gel:CNFs 75:25, 50:50 and 25:75, followed by modulating their stability by cross-linking using dehydrothermal treatment (DHT) in simulated body-like conditions.

DHT treatment creates bridges between molecules and decreases the degradation rate. It is a physical treatment that removes water from polymer molecules thanks to the increased temperature (160 °C) and vacuum conditions. This results in the formation of intermolecular cross-links through condensation reactions, either by esterification or by amide formation [8]. DHT treatment is preferable to other cross-linking methods, as it does not involve the use of cytotoxic reagents [9,10]. Gel was chosen for its extensively proven biomimicry with the extracellular matrix (ECM), thanks to its derivation from collagen, together with its versatile and reactive chemical structure [11], while the biocompatibility of CNFs, their exceptional mechanical properties and surface area, enlighten them as an ideal additive to meet the requirements, in combination with gelatin, for the design of scaffolds addressing soft-tissue engineering [12,13,14,15]. Through the tuning of blending and cross-linking processes, the components were combined to create composites with improved physical-chemical and mechanical properties, together with an enhanced bioactivity compared to the solitary raw materials [16]. The paper aims to prove the effectiveness of the Gel:CNF blending and cross-linking process and its synergistic effect on the functional and mechanical properties compared to the mere sum of the individual components. An effective strategy is outlined to develop biocompatible and bioresorbable 3D porous scaffolds as a promising option for in situ soft-tissue engineering with easily tunable properties.

## 2. Materials and Methods

### 2.1. Biopolymers

Cellulose nanofibrils (CNFs) produced from never-dried bleached softwood pulp fibers were used as the material source. The preparation was performed using the 2,2,6,6-tetramethyl-piperidine-1-oxyl radical (TEMPO) mediated oxidation, as described by Saito et al. in 2006 [17]. Then, 1.9 mmol of NaClO per gram of cellulose was used for the oxidation reaction. Fibrillation was done by using a Rannie 15 type 12.56× homogenizer (APV, SPX Flow Technology, Silkeborg, Denmark), with a pressure drop of 600 bar in the first step and 1000 bar in the second step. The CNF suspension had a solid content of 1.1%. The carbonyl group content was determined using conductometric titration as previously described [18,19]. The equipment used was a 902 Titrando, an 856 conductivity module and Tiamo software (Metrohm, Herisau, Switzerland).

Gelatin (Gel) was received from Italgelatine (Cuneo, Italy). It was extracted from pig skin and produced with mesh 4 and bloom 280.

### 2.2. Synthesis Process

**Figure 1:**
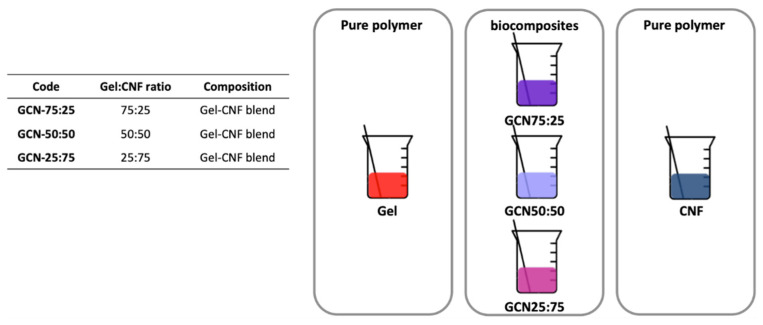
Representation of gelatin (Gel) and cellulose nanofibrils (CNFs) ratio in the developed blends.

Biopolymer blends with a final concentration of 1.1 wt% were obtained by mixing an aqueous solution of Gel (1.1 wt%) and an aqueous suspension of CNFs (1.09 ± 0.01 wt%) in order to obtain Gel:CNF weight ratios of 75:25, 50:50, 25:75, respectively. In detail, 25 g of Gel solution was prepared by dissolving gelatin in water at 45 °C under magnetic stirring. In order to obtain the Gel:CNF weight ratio of 75:25, 75 g of the CNF suspension was added to the Gel solution and mechanically mixed for an hour as shown in Figure 1. Similarly, 50 g and 75 g of CNF suspension were added to 50 g and 25 g of the Gel solution in order to obtain the Gel:CNF weight ratios of 50:50 and 25:75, respectively. To obtain the 3D porous structures, the blends were poured in Teflon molds of Ø = 8 mm and freeze-dried. The freeze-drying cycle included a controlled freezing ramp of 50 °C/h until −40 °C and two heating ramps, the first of 5 °C/h from −40 °C to −5 °C and the second 3 °C/h until 20 °C for about three days under vacuum conditions (*p* = 0.086 mbar). Finally, the obtained biocomposites were cross-linked by DHT performed in an oven at 160 °C, with a pressure of 0.01 mbar, for 48 h [20,21,22].

### 2.3. Morphological Characterization

Biocomposite morphology was evaluated after freeze-drying by environmental scanning microscopy (ESEM, Quanta 200 FEG, FEI Company, Hillsboro, OR, USA). The samples were prepared by fixing them onto aluminum stubs using carbon tape, and then Au coated using the coating unit Polaron Sputter Coater E5100 (Polaron Equipment, Watford, Hertfordshire, UK).

Biocomposite viscosity was assessed by means of a rotation rheometer (C-VOR 120, Bohlin Instruments, Malvern, UK) with a cone/plate CP40/4° (Ø = 40 mm, angle = 4°, working distance = 150 µm) geometry for a shear profile definition among 0.05 Pa and 100 Pa, with a sweep time of 300 s. A solvent trap was used to avoid any water evaporation during the test, despite the whole analysis being performed at 25 °C.

Biocomposite porosity was determined by means of two different methods. A density method was used to determine their total porosity, using the biocomposite weight and volume data to calculate the density and consequently the porosity rate [12,23]. Biocomposite density (*ρ*) was calculated through the following equation (Equation (1)):

(1) $$\rho =\frac{W}{\pi \times {\left(\frac{D}{2}\right)}^{2}\times H}$$

where *W* was the biocomposite weight, *D* the diameter and *H* the height. The obtained density was divided by the materials’ theoretical density, determined by the respective weight fraction (*X_i_*) of each component and their theoretical density (Equation (2)):

(2) $$\rho_{theoretical}=\left(\rho_{theoretical\;a}\times X_{a}\right)+\left(\rho_{theoretical\;b}\times X_{b}\right)$$

Finally, the total porosity was calculated through the following equation (Equation (3)):

(3) $$Total\;porosity_{\%}=100-\left(\frac{\rho }{\rho_{theoretical}}\times 100\right)$$

Three replicates were assessed (*n* = 3) and the results are expressed as mean ± standard error.

Water-squeezing method was used to determine the water amount absorbed in the biocomposite before and after the manual squeezing [12,24]. Squeezing tests were performed by soaking samples in phosphate buffered saline (PBS) under shaking at room temperature. After 1 h, the samples were weighted (*M_swollen_*), and manually squeezed on filter paper to easily remove the water. The filter paper was changed until no water was detected around the samples. After that, the samples were weighted again (*M_squeezed_*). Since the water in the macropores would be released easier than the water in the micropores, an evaluation of the macropores’ volume percentage could be made through the following equation (Equation (4)):

(4) $$Macropores\;volume_{\%}=\frac{\left(M_{swollen}-M_{squeezed}\right)}{M_{swollen}}\times 100$$

Three replicates were assessed (*n* = 3) and the results are expressed as mean ± standard error.

### 2.4. Chemical-Physical Characterization

The interaction degree between the Gel and CNFs and the cross-linking degree due to DHT were evaluated by TNBS (2,4,6-trinitrobenzenesulfonic acid) assay, previously reported by Balakrishnan et al. [25]. TNBS test spectrophotometrically evaluated the amount of free primary amines (–NH_2_) of the Gel that decreased after blending with the CNFs and also after DHT treatment, due to the reaction between their functional groups, and measured the efficiency of the interaction degree (Int%) as well as the cross-linking degree (CD%).

Briefly, to perform Int% tests, 1 mL of NaHCO_3_ solution (4% *w*/*v*) was added to each 5 mg of biocomposites and Gel, respectively. After 30 min, a freshly prepared TNBS (0.5% *w*/*v*) solution was added. The reaction mixture was heated at 40 °C for 2 h and then 3 mL of 6 M HCl were added. The reaction mixture was heated at 60 °C for 90 min and then diluted 1:1 with milliQ water and cooled to room temperature. The absorbance at 415 nm was recorded using a UV–vis spectrophotometer (Perkin-Elmer Lambda 35, Milano, Italy). For each sample, the measurements were run in triplicates and a biocomposite-free blank was prepared under the same conditions. The interaction degree (*Int%*) was evaluated through the following equation (Equation (5)):

(5) $$Int_{\%}=\left(1-\frac{Biocomposite\;absorbance}{Gelatin\;absorbance}\right)\times 100$$

Three replicates were assessed (*n* = 3) and the results are expressed as mean ± standard error.

Briefly, to perform the CD% tests, 1 mL of NaHCO_3_ solution (4% *w*/*v*) was added to 5 mg of cross-linked biocomposites and non-cross-linked biocomposites. The TNBS assay was performed as described above. For each sample, the measurements were run in triplicates and a biocomposite-free blank was prepared under the same conditions. The cross-linking degree (*CD%*) was evaluated through the following equation (Equation (6)):

(6) $$CD_{\%}=\left(1-\frac{Cross-linked\;biocomposite\;absorbance}{Not\;cross-linked\;biocomposite\;absorbance}\right)\times 100$$

Three replicates were assessed (*n* = 3) and the results are expressed as mean ± standard error.

Swelling tests were performed by soaking the samples in a phosphate buffered saline (PBS) solution with 0.1% *w*/*v* of NaN_3_, incubated at 37 °C under shaking [12,24]. At regular time intervals, the gels were weighed, after letting them rest for some seconds on a non-absorbent surface. The swelling ratio (*S_r_*) was calculated through the following equation (Equation (7)):

(7) $$S_{r}=\frac{W_{s}-W_{d}}{W_{d}}$$

where *W_s_* was the swollen sample weight and *W_d_* the dry sample weight before soaking.

Three replicates were assessed (*n* = 3) and the results are expressed as mean ± standard error.

The degradation of the scaffolds was measured through a weight-loss test. It was performed by soaking the samples in a phosphate buffered saline (PBS) solution with 0.1% *w*/*v* of NaN_3_ at 37 °C while shaking [12,26]. At regular time intervals, the gels were taken out from the medium, washed three times in milliQ water and freeze-dried. The samples were then weighed and the weight loss (%) was calculated through the following equation (Equation (8)):

(8) $$Weight\;loss_{\%}=\frac{W_{i}-W_{f}}{W_{f}}\times 100$$

where *W_i_* was the dried sample’s initial weight while *W_f_* was the freeze-dried sample weight at a specific time point.

The static contact angle was determined to evaluate the solid–liquid interfacial tension of the materials. Water affinity was tested using a static contact angle test on the materials in a non-porous film-like form, produced by casting wet materials on microscope slices before drying at room temperature. For the test, 1 μL of distilled water was added to the film surface and the drops static contact angle was measured using a tensiometer (Video-Based Optical Contact Angle Meter OCA 15+, Innovent, Filderstadt, Germany). Values were expressed as mean ± standard error (*n* = 10).

### 2.5. Mechanical Characterization

The mechanical properties were evaluated using a dynamic mechanical analyzer Q800 (TA Instruments, Milano, Italy) in compression mode. Measurements were carried out at 37 °C and the samples (Ø = 7–8 mm, h = 4–6 mm) were tested wet after immersion in PBS overnight (Sigma Aldrich, Saint Louis, MO, USA) at 37 °C while shaking. Before the measurement, a preload force was applied to the samples to ensure that the entire biocomposite surface was in contact with the compression plates. The Young’s modulus was evaluated by means of a stress–strain test after an isothermal period of 5 min at 37 °C with a force ramp rate of 0.5 N/min until 8 N [12,24]. The slope of the linear fit was calculated in a strain range from 0 to 10%. Results were reported as the average of five tested samples.

A multi-frequency test was performed to assess the viscoelastic behavior of the samples [12,27]. The spectra were obtained from a frequency scan from 0.1 to 10 Hz under a constant strain amplitude of 75 µm. Results were reported as the average of five tested samples.

To evaluate the linearity zone of the samples, the creep test was performed in the range between 0.001 and 0.1 MPa. A stress of 0.01 MPa was chosen as suitable for the actual creep test [12,28]. After an isothermal period of 5 min at 37 °C, each sample underwent a 15 min compression at a defined and constant stress of 0.01 MPa before a 15 min rest to evaluate the recovery rate. The results were reported as the average of three tested samples.

### 2.6. Cell Culture

Human mesenchymal stem cells (hMSCs) purchased from Lonza (Basel, Switzerland), were cultured in a standard medium constituted by α-modified Eagle’s medium (α-MEM, Gibco), penicillin–streptomycin (100 U/mL–100 µg/mL), 15% fetal bovine serum (FBS) and Fibroblast growth factor(FGF)-basic (10 ng/mL). Cultured cells were kept at 37 °C in an atmosphere of 5% CO_2_. Cells were detached by trypsinization and centrifuged, and the cell number and cell viability were assessed with a trypan blue dye exclusion test. The biocomposites were sterilized by 25 kGy γ-ray irradiation and placed in a 24-well plate for pre-soaking into a culture medium for 24 h. The cells seeding process onto the biocomposites was done by carefully adding 30 μL cell suspension (5.0  ×  104 cells). The cells were seeded on the upper surface of the biocomposites letting them settle for 30 min before adding 1.5 mL of cell culture medium per well. The medium was changed every 3 days. All cell handling procedures were performed in a sterile laminar flow hood. All incubation steps were performed at 37 °C with 5% CO_2_.

### 2.7. Cell Viability Assay

Cell viability was tested quantitatively using a 3-(4,5-dimethylthiazol-2-yl)-2,5-diphenyltetrazolium bromide (MTT) assay. MTT (5 mg/mL) was dissolved in PBS buffer 1x, then, at each time point (1, 3 and 7 days), the solution of MTT was added in proportion 1:10 to the cell samples. After 2 h of incubation at 37 °C and 5% CO_2_, the biocomposites were transferred into tubes containing 1 mL of dimethyl sulfoxide (DMSO) that dissolved formazan crystals. After a brief centrifugation, 200 µL of the supernatant was transferred into a 96-well plate and absorbance read at 570 nm using a Multiskan FC Microplate Photometer (Thermo Scientific, Monza, Italy). The registered absorbance was directly proportional to the number of metabolically active cells. For each time point, two samples were used and analyzed in technical triplicate. Qualitative cell viability was assessed using the Live/Dead assay kit (Invitrogen, Monza, Italy). After 1 day of culture, a sample for each biocomposite composition was washed with PBS 1x for 5 min and incubated with 2 μM calcein acetoxymethyl (calcein AM) plus 4 μM ethidium homodimer-1 (EthD-1) for 15 min at 37 °C in the dark. Samples were rinsed in PBS 1x and the images were acquired by an inverted Ti-E fluorescence microscope (Nikon, Firenze, Italy).

### 2.8. Cell Morphology Analysis

Cellular morphology was observed via SEM observations. Briefly, after 3 days of culture, the samples were washed with 0.1 M sodium cacodylate buffer at pH 7.4 and then fixed in a solution of 0.1 M sodium cacodylate containing 2.5% glutaraldehyde for 2 h at 4 °C. The samples were washed again with 0.1 M sodium cacodylate buffer and freeze-dried. The dehydrated samples were sputter coated with gold and observed using a Quanta scanning electron microscope (ESEM Quanta 200, FEI, Company, Hillsboro, OR, USA).

### 2.9. Statistical Analysis

The MTT assay results were expressed as mean ± standard error of the mean (SEM) plotted on a graph. Statistical analyses were performed by the GraphPad Prism software (version 6.0, GraphPad Software Inc., San Diego, CA, USA). Statistical significances between the samples were calculated using the two-way analysis of variance (ANOVA) test.

## 3. Results and Discussion

### 3.1. Biocomposites Preparation

The blending processes were designed to combine the best properties of the two selected polymers in order to obtain biocomposites with improved mechanical performances without losing their biocompatibility, chemical stability and flexibility. In this work, three blend compositions of Gel and CNFs were studied as reported in Figure 1. Gel was chosen due to its biocompatibility and because of its similarity with collagen as the main component of the extracellular matrix. However, Gel has poor mechanical properties (i.e., stiffness), lacks the fibrous character of collagen and is characterized by a very fast degradation rate. To face these weak points, different amounts of CNFs were added, since the fibrils have high stiffness, fibrous character and long degradation time together with a well assessed biocompatibility [29,30,31]. The blends were prepared by varying the polymer ratio (Gel:CNFs) while the final total polymer weight in water was kept constant (1.1 wt%). The blends were freeze-dried and cross-linked using the DHT treatment at 160 °C under vacuum to promote the creation of bridges between the polymer chains and to modify their degradation rate and mechanical performances [9].

### 3.2. Morphological Characterization

**Figure 2:**
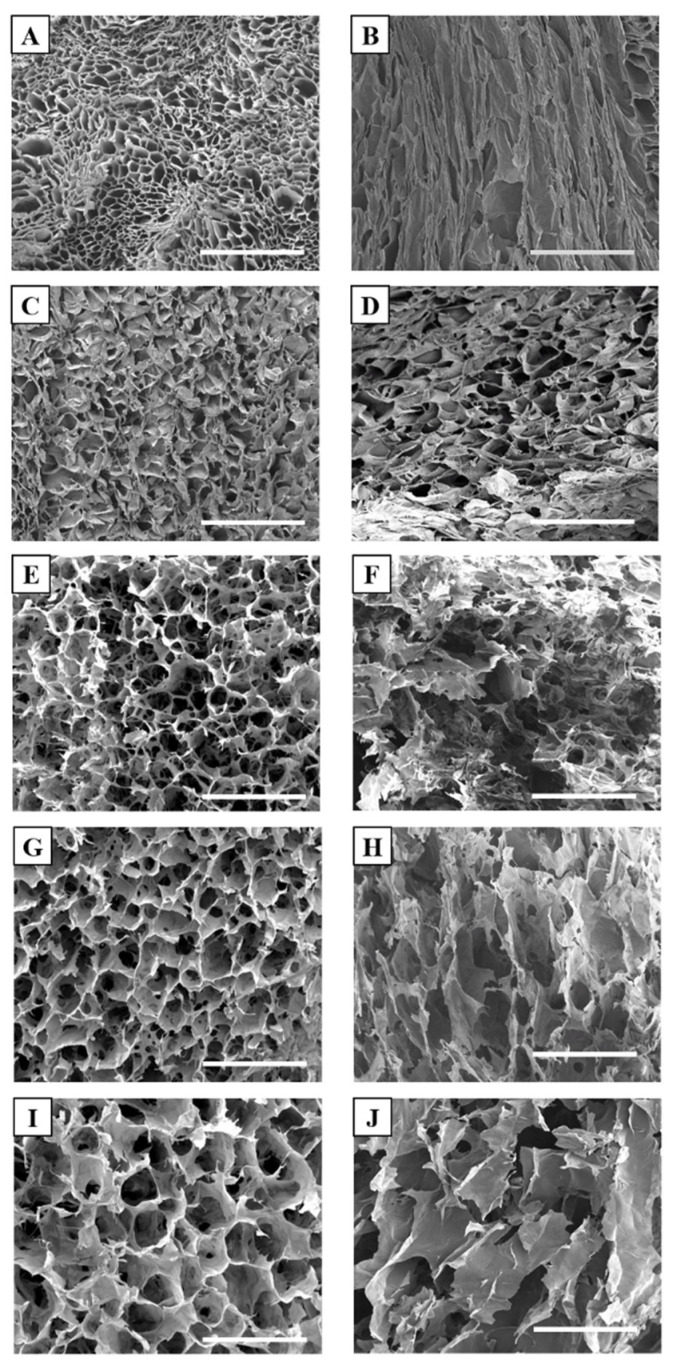
Cross- and sagittal-sectioned SEM images of (**A**,**B**) Gel; (**C**,**D**) CNFs; (**E**,**F**) GCN(Gel:CNFs)-75:25; (**G**,**H**) GCN-50:50; (**I**,**J**) GCN-25:75, all cross-linked by dehydrothermal treatment (DHT). Scale bars are 500 µm.

**Figure 3:**
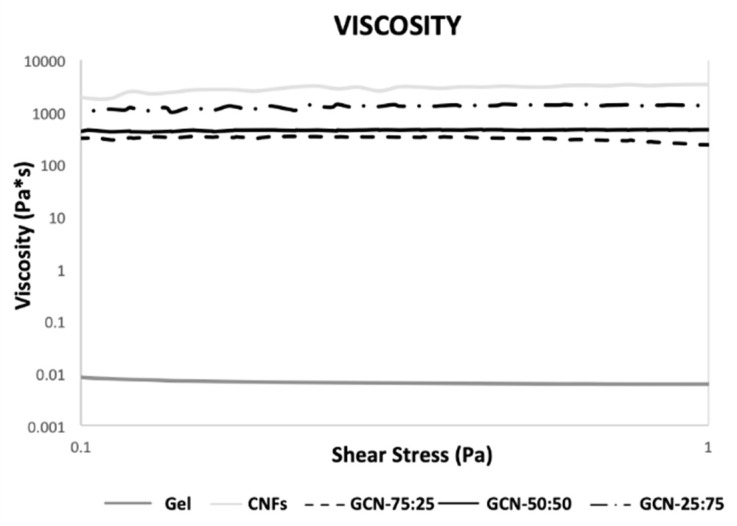
Viscosity behavior of the biocomposites and the pure materials.

Pore morphology and distribution play a crucial role since they affect: (i) the cellular adhesion, proliferation and growth; (ii) the permeation of nutrients and oxygen for the cells from the surface towards the core of the scaffold and the elimination of CO_2_ and other metabolites from the core towards the surface; and iii) the mechanical behavior of the whole scaffold. ESEM images (Figure 2E–J of the prepared blends show similar pore sizes and pore shape: the similarity in the pore size is due to the fact that porosity is steered by the freeze-drying process which in turn is controlled by the size and geometry of the ice crystals formed during the freezing process [32]. On the other hand, the pore size of the blends is larger than the ones of the pure polymers (Gel, CNFs) (Figure 2A–D). The different aspect ratio of pores between the blends and the pure polymers can be assigned to their different viscosities. The viscosities of pure polymers and blends are reported in Figure 3 [33]. The low viscosity of Gel is responsible for the mono-directional growth of ice crystals, inducing the formation of a channel-like porous structure (Figure 2B), with a channel diameter of about 50–100 µm. In the case of CNFs, its high viscosity promotes the formation of small randomly oriented ice crystals (Figure 2D). When CNFs are added to Gel, the ice crystal formation during the freeze drying is retarded and hampered by the increase in the blend viscosity, thus the direction of ice crystals growth becomes more isotropic, and the derived porosity is randomly oriented and characterized by bigger pores (100–250 µm) (Figure 2F,H,J) [34].

However, despite modifying the Gel:CNF ratio, all the biocomposites showed rough surfaces and a porous structure with interconnected pores homogeneously distributed [10,12] with sizes ranging from less than 100 up to 250 µm, suitable for cell colonization and proliferation.

Porosity was also evaluated by density measurements and water-squeezing methods, as reported in Table 1. Porosity tests, according to the SEM images, show high porosity over 90% for all the samples due to the high-water content before lyophilization. The lower value of porosity registered for CNFs was due to the inter-fibril linkages, generating a tight structure. Different polymer ratios did not significantly affect this parameter, but all blends generally showed slightly higher porosity compared to the pure polymers (Gel or CNFs). This behavior is affected by the interactions between the polymers, creating a well dispersed blend able to self-assemble entrapping water molecules [24].

Using the water-squeezing method, the porosity of the pure polymers was lower due to the high number of smaller pores trapping the inner water that could not be released by simply squeezing (Table 1). The difference between the Gel and CNF macro porosity is reflected in the different density observed by the squeezing method and confirms the denser structure of CNFs. CNFs have small rounded-shaped pores whereas Gel has elongated ellipse-like pores with sagittal axis bigger than the transversal axis. The different shapes of the pores and the different pore volumes resulted in lower macro porosity for the CNFs compared to Gel.

### 3.3. Chemical-Physical Characterization

The TNBS assay, spectrophotometrically determining the amount of unreacted free amines in the analyzed samples (Table 2), was used to measure the Gel–CNF interaction degree (Int%) and the efficiency of cross-linking degree (CD%) [35].

By comparing the blends and pure Gel, it is possible to determine the degree of interaction between the Gel and CNFs during the blend formation. On the other hand, by comparing the cross-linked blends with non-cross-linked ones, it is possible to determine the cross-linking degree due to DHT treatment.

The results (Table 2) demonstrate that when adding CNFs to Gel the Int% increases, meaning that the number of free amines is reduced as expected. When DHT cross-linking is applied, the efficiency of the procedure is influenced by the interference with CNFs.

**Scheme 1:**
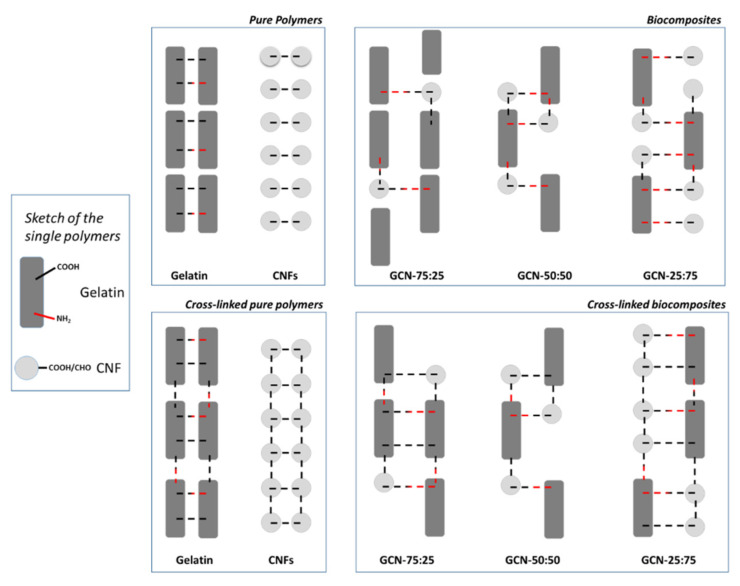
Schematic illustration of the possible interaction between polymers during blending process (top scheme) and cross-linking process (down scheme). Note that the condensation reactions can occur between carboxylic groups (black bonds in Gel and CNFs), carbonyl groups (black bonds in CNFs) and amino groups (red bonds in Gel).

Polymer ratio affects the covalent linkage formation, but not in a linear way. In fact, in GCN (Gel:CNFs)-50:50 the CD% is lower than for the GCN-75:25 and the cross-linking seems hampered by the presence of CNFs (Scheme 1) [9,12,36]. However, when the CNF content increases from 50% to 75%, the cross-linking degree increases. This is not easy to understand but the explanation probably lies in the relative amounts of functional groups and the proximity between them. Xuefei et al. [37] demonstrated that DHT cross-linking of collagen resulted in formation of both covalent and hydrogen bonding. The bonding between the peptide chains seems to be prevalent, as it is proven by the reduction in the number of free acidic and basic residues on collagen molecules after the DHT treatment. They also proved that after DHT cross-linking, both non-covalent and covalent chemical bonds co-exist, and improve the properties compared to the non-cross-linked material. According to Yannas et al. [38] these chemical bonds can be formed by condensation, and also by esterification and amidation between carboxylic and amine groups. Thanks to the chemical structure of Gel and CNFs, presenting this kind of functional groups, all the aforementioned reactions can also occur within our GCN biocomposite.

In detail, the CNFs used in the study have an aldehyde content of 211 ± 60 µmol/g and carboxylic acids of 764 ± 60 µmol/g, while the gelatin used was Type A gelatin, reported in literature to have 800 µmol/g carboxylic acid groups and a primary amine content of 286 µmol/g [39]. Primary amines and aldehyde groups react easily with each other in a click reaction if they are close enough, but as described, after the DHT all functional groups are involved in cross-linking reactions, so the key point is the distance between the polymer chains/fibrils. From the results given in Table 2, it is evident that there is no significant difference between the GCN-75:25 and GCN-25:75, while a significant difference is noted for sample GCN-50:50. If we consider the possible distance between the polymers/fibrils we can probably assume that the distance between the Gel chains and nanofibrils are larger in the GCN-50:50 sample than between the Gel chains in the GCN-75:25 sample and the nanofibrils in the GCN-25:75 sample. Thus, when the Gel:CNF ratio is unbalanced, more functional groups are settled in a suitable distance to cross-link. Cellulose nanofibrils have a large tendency to self-assemble and form strong bonds with neighboring fibrils. If the water is involved, this phenomenon is called hornification, and the maximum effect is with a water content of approximately 0.25 g water/g cellulose [40]. The self-assembling effect is reported to occur even if large amounts of another component are added, e.g., up to 60% maltodextrin [41]. It is likely that the self-assembling of CNFs, strengthened by DHT, is the main mechanism in the GCN-25:75 sample.

Scheme 1 illustrates the possible links between the two polymers as well as the tentative linkages due to DHT cross-linking and is used as a simple way to visualize and describe the nonlinear variation of CD% in the blends.

The evaluation of the cross-linking extent demonstrated that, by blending and cross-linking different polymers, it was possible to engineer new biocomposite blends, capable of different chemical interactions and characterized by tunable physical-chemical properties.

Blend hydrophilicity was measured by static water contact angle measurement on films made from blends and the results are reported in Table 3. Some significant changes in the contact angles were recorded among the samples, and all of them showed contact angles higher than 70°, (higher with respect to the data found in the literature) indicating a less hydrophilic behavior. As previously reported, this is due to the cross-linking treatment, which reduces the available hydroxyl and amino groups of the polymers (Gel and CNFs), causing the formation of covalent linkages between them [21,42,43].

**Figure 4:**
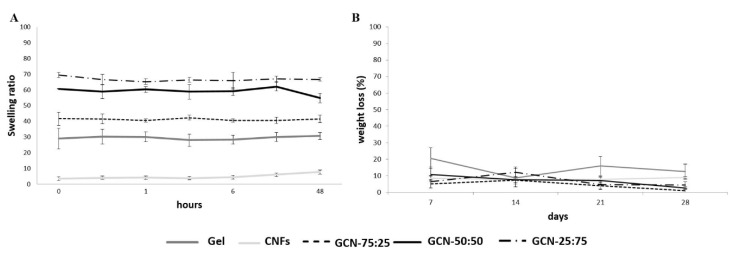
(**A**) The swelling behavior; and (**B**) the biocomposite stability in wet conditions in PBS at 37 °C of the Gel–CNF biocomposites.

The swelling test was carried out to evaluate the capacity of the biocomposites to absorb water and consequently cell medium, which is essential for cell colonization. Each sample showed a distinctive water uptake, influenced by the compositional and morphological differences as illustrated in Figure 4A. When observing the swelling behavior, a rapid water uptake during the first hour of experiment was clear for the all samples maintaining the same saturation level up to 48 h.

Comparing the different samples, the swelling of all blends was higher than for pure polymers (Gel and CNFs); i.e., the higher values of hydrophilicity and porosity cause a larger degree of swelling of the samples. These data are in contrast with the measured values of contact angles where the porosity was not considered, because the tests were carried out on dried and non-porous films. However, swelling is mainly affected by the porosity of the structure; therefore, the blends showed higher swelling due to a higher porosity than the pure polymers, as already evaluated through the water squeezing method and ESEM microscopy [43].

Among blends, higher amounts of CNFs caused a larger swelling, as is in agreement with ESEM microscopy, where a slightly higher pore growth was observed in the GCN-25:75 sample compared to GCN-75:25. Finally, the swelling was fast and did not change during incubation time, meaning that no initial degradation and no loss of structure occurred.

The weight loss test was carried out to evaluate the efficacy of DHT that is essential for maintaining the shape and slowing down the degradation rate of the scaffold when in vivo [9]. As reported in Figure 4B, after 28 days at 37 °C in PBS, all samples lost 10–20% of their overall weight, macroscopically preserving their shape and structure. The degradation rate is a crucial point in the choice of the scaffolding strategies, because a too fast degradation would not support the tissue remodeling process [12]. A compromise between the tissue growth and the biocomposite degradation rate is essential to guarantee a sustainable scaffold throughout the cellular regenerative process [44,45,46].

### 3.4. Mechanical Characterization

**Figure 5:**
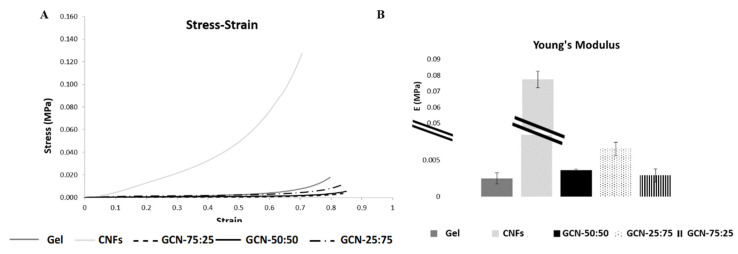
Compressive mechanical test of Gel–CNF biocomposites after soaking in phosphate buffered saline (PBS) at 37 °C overnight. (**A**) Average stress-strain curves (*n* = 5 specimens); (**B**) Young’s modulus calculated as the slope of the stress-strain curve in the strain range of 0.01–0.10 (*n* = 5 specimens).

The mechanical behavior of the blends was evaluated in simulated body fluids since the biocomposites were expected to be used in a hydrated environment. Figure 5 shows the mechanical properties of cross-linked biocomposites in comparison to the pure polymers, evaluated under compression mode. Pure polymers showed a very different Young’s modulus, higher for CNFs compared to Gel (0.0774 MPa versus 0.0025 MPa) confirming the use of CNFs for nano-reinforcement. The blends show similar mechanical properties; GCN-25:75 shows slightly higher Young’s modulus (0.0065 MPa), but far from the one of pure CNFs. When the CNF amount in the blend is reduced, Young’s modulus decreases in a linear way passing from 0.0036 MPa to 0.0029 MPa for GCN-50:50 and GCN-75:25 (Figure 5B) [36,47].

**Figure 6:**
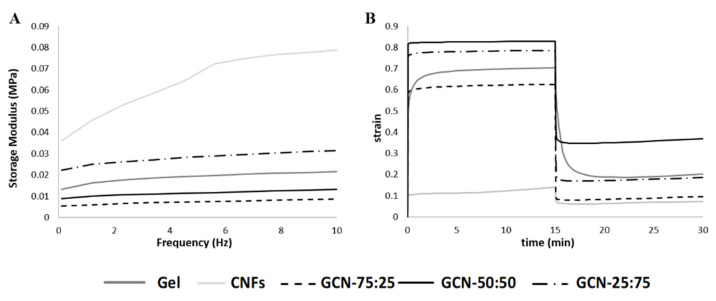
Compressive mechanical test of the Gel–CNF biocomposites previously immersed in PBS at 37 °C overnight. (**A**) The average storage modulus (E’)-frequency curves from 0.1 Hz to 10 Hz (*n* = 5); and (**B**) the average creep curves obtained in a creep time of 15 min and a recovery time of 15 min (*n* = 5).

Figure 6A shows the viscoelastic properties of the biocomposites when they were subjected to different frequencies in the range 0.1–10 Hz. Specific frequencies were chosen to simulate in vivo stress conditions, evaluating the biocomposites’ behavior in term of storage (E’) and loss (E’’) modulus [48,49]. E’’ are not reported because the values observed were very low in comparison to E’, indicating a predominantly elastic behavior [48]. For all the biocomposites, the storage modulus (E’) did not show significant differences by varying the frequency, except for the CNFs where E’ increased from 0.0359 MPa to 0.0786 MPa. Although the storage modulus (E’) does not significantly change along with frequency, it changes depending on the Gel:CNF ratio. In agreement with the stress–strain data, the CNF sample displayed the highest storage modulus E’, and accordingly, the lowest values were obtained in the blends with a lower CNF amount. In detail, GCN-75:25 and GCN-50:50 revealed a storage modulus E’ of 0.005 MPa and 0.009 MPa, respectively. Only GCN-25:75, due to its higher amount of CNFs, revealed a storage modulus (0.0221 MPa) between the one of pure CNFs and Gel (0.0132 MPa).

Finally, a creep test was performed to evaluate the materials’ behavior when subjected to high stress for a certain time [12]. The preliminary linearity study (data not showed) allowed to identify the suitable stress value to apply on all samples. Creep tests (Figure 6B) showed that all the biocomposites, after being subjected to a static stress of 0.01 MPa for 15 min, can recover almost all their shape, showing an elastic behavior. Compared to the pure polymers, which displayed a strain recovery of 71% (Gel) and 59% (CNFs), only GCN-50:50 revealed a lower strain recovery of about 55%. Contrariwise, GCN-25:75 and GCN-75:25 highlighted a good strain recovery, higher than the pure materials (85% and 84%, respectively).

### 3.5. Evaluation of Biological Performance of Biocomposites

**Figure 7:**
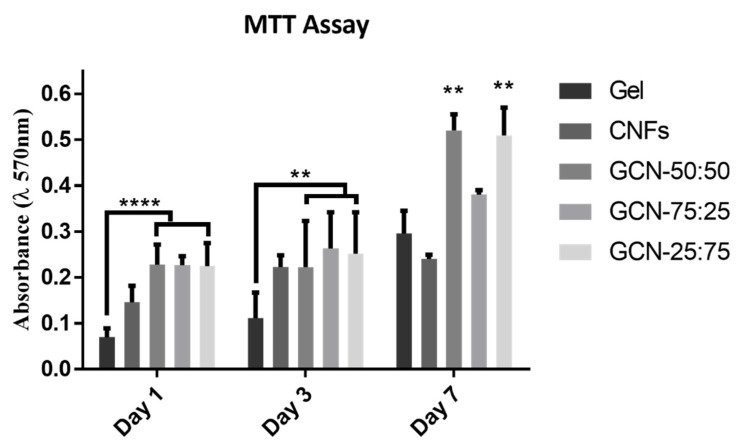
Quantitative cell viability analysis performed with MTT assay. Absorbance registered at 570 nm is directly related to the number of metabolically active cells. Time points at 1, 3 and 7 days after seeding. For each time point **** *p* ≤ 0.0001; ** *p* ≤ 0.01.

In order to evaluate the biomaterials’ cytotoxicity, both quantitative and qualitative cell viability tests were performed. The quantitative viability test (MTT assay) showed for all the biomaterials an overall increment of absorbance over time, directly related to the quantity of metabolically active cells (Figure 7). GCN-50:50 and GCN-25:75 achieved the highest cell viability values at day 7, showing a significant statistical difference compared with all the other biocomposites (** *p* ≤ 0.01). The biocomposites had in general a higher cytocompatibility compared to the pure materials (Gel, CNFs), with a significant difference of *p* ≤ 0.0001; ** *p* ≤ 0.01 at day 1 and 3, respectively.

**Figure 8:**
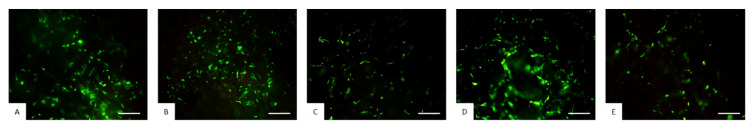
Cell viability analysis performed with the Live/Dead kit where the calcein acetoxymethyl (calcein AM) labels the living cells in green and ethidium homodimer-1 labels dead cells in red. The HMSCs seeded on (**A**) Gel; (**B**) CNFs; (**C**) GCN-50:50; (**D**) GCN-25:75; (**E**) GCN-75:25—images taken 1 day after seeding. Scale bar: 200 µm.

The qualitative viability test was performed using Live/Dead kit one day after cell seeding (Figure 8). Here, the living cells are labelled in green and the necrotic calls are labelled in red.

**Figure 9:**
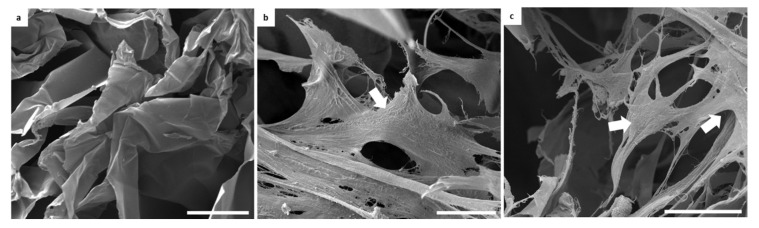
SEM observations of (**a**) the unseeded GCN-25:75 and (**b**,**c**) the cell-seeded GCN-25:75. Images taken 3 days after seeding. White arrows pointing at cells attached on the biomaterial surface. Scale bar: (**a**,**b**) 25 µm, (**c**) 50 µm.

For all biocomposites, the green-labeled cells were qualitatively higher in ratio compared to the red-labeled cells, which were almost not detected. This indicates a lack of cytotoxicity. The morphology of the cells cultured on the most promising biocomposites was analyzed through SEM observations. The analysis conducted at day 3 after cell seeding, showed anchored and healthy cells growing on the GCN-25:75, as their stretched cytoskeletons testify (Figure 9).

In conclusion, this work well defines the key role of blends of gelatin and CNFs. All the discussed characterizations demonstrate that properties of the Gel–CNF blends are not the result of a physical mixing characterized by intermediate features only. In fact, amino functional groups of Gel react with aldehydic or carboxylic functional groups on CNFs through covalent linkages capable of generating interesting blends with new and different features [50]. Especially, by changing the Gel:CNF ratio, it is possible to modulate the morphological, chemical and mechanical features of the biocomposites. This is essential for the creation of different biomaterials with tunable properties, and capable to guide and favor cells processing to regenerate or form new tissues [27,51,52]. Although in vitro tests are only at a preliminary stage and are not able to evaluate a specific differentiation depending on Gel:CNF ratio, they revealed that each blend creates a different microenvironment in which the cells can grow and proliferate [53,54,55].

## 4. Conclusions

New gelatin-based polymeric biocomposites for tissue regeneration were successfully designed, developed and tested. The addition of cellulose nanofibrils to the gelatin matrix was selected to improve the mechanical performance of the resulting biocomposites. By blending the gelatin and CNFs in different ratios and applying DHT cross-linking processes, reinforced biocomposites with isotropic porosity, the ability of controlled swelling and degradation rate in physiological conditions were obtained. Besides, the biocomposites showed elastic behavior together with the ability to recover almost all of their shape, when subjected to a static stress.

Our results proved that all the Gel–CNF biocomposites have advanced properties compared to the native materials, gelatin and CNFs, thanks to the synergistic interaction between them, e.g., they were better in promoting cell adhesion, colonization and proliferation.

In conclusion, the creation of new materials with tunable physical-chemical, morphological and mechanical properties opens new perspectives in tissue engineering, based on the modulation of a variety of cues affecting the hosting cells’ behavior and fate.

## Figures and Tables

**Table 1:** Summary of porosity for the Gel–CNF biocomposites.

Samples	Porosity through Density Method	Porosity through Water-Squeezing Method
Gel	98.0 ± 0.5%	87.0 ± 1%
CNFs	94.0 ± 0.5%	48.0 ± 4%
GCN-75:25	99.0 ± 0.1%	96.0 ± 0.2%
GCN-50:50	99.0 ± 0.1%	96.0 ± 0.1%
GCN-25:75	99.0 ± 0.1%	94.0 ± 1%

**Table 2:** Interaction degree for the polymer chains (Int%) and the cross-linking degree (CD%) after the DHT treatment for the Gel–CNF biocomposites measured by TNBS (2,4,6-trinitrobenzenesulfonic acid) assay.

Samples	Int% Measured before DHT	CD% Measured after DHT
	(Blank = Gel)	(Blank = Uncross-Linked Biocomposite)
GCN-75:25	5 ± 1%	48 ± 3%
GCN-50:50	16 ± 3%	22 ± 2%
GCN-25:75	49 ± 2%	56 ± 2%

**Table 3:** Summary of the contact angles of the cross-linked pure polymers and the Gel–CNF biocomposites.

Cross-Linked Samples	Contact Angles
Gel	88.9 ± 6.6°
CNFs	72.8 ± 2.4°
GCN-75:25	94.1 ± 2.8°
GCN-50:50	99.8 ± 2.2°
GCN-25:75	87.7 ± 3.5°
